# One-Shot Learning With Attention-Guided Segmentation in Cryo-Electron Tomography

**DOI:** 10.3389/fmolb.2020.613347

**Published:** 2021-01-12

**Authors:** Bo Zhou, Haisu Yu, Xiangrui Zeng, Xiaoyan Yang, Jing Zhang, Min Xu

**Affiliations:** ^1^Department of Biomedical Engineering, Yale University, New Haven, CT, United States; ^2^Computational Biology Department, Carnegie Mellon University, Pittsburgh, PA, United States; ^3^Computer Science Department, University of California, Irvine, Irvine, CA, United States

**Keywords:** one shot learning, cryo-ET, macromolecule classification, macromolecular segmentation, attention

## Abstract

Cryo-electron Tomography (cryo-ET) generates 3D visualization of cellular organization that allows biologists to analyze cellular structures in a near-native state with nano resolution. Recently, deep learning methods have demonstrated promising performance in classification and segmentation of macromolecule structures captured by cryo-ET, but training individual deep learning models requires large amounts of manually labeled and segmented data from previously observed classes. To perform classification and segmentation in the wild (i.e., with limited training data and with unseen classes), novel deep learning model needs to be developed to classify and segment unseen macromolecules captured by cryo-ET. In this paper, we develop a one-shot learning framework, called cryo-ET one-shot network (COS-Net), for simultaneous classification of macromolecular structure and generation of the voxel-level 3D segmentation, using only one training sample per class. Our experimental results on 22 macromolecule classes demonstrated that our COS-Net could efficiently classify macromolecular structures with small amounts of samples and produce accurate 3D segmentation at the same time.

## 1. Introduction

Cryo-Electron Tomography (cryo-ET) has made possible the observation of cellular organelles and macromolecular structures at nano-meter resolution with native conformations (Lučić et al., 2013). Without disrupting the cell, cryo-ET can visualize both known and unknown cellular structures *in situ*^1^ and reveals their spatial and organizational relationships (Oikonomou and Jensen, 2017). Using cryo-ET, it is possible to capture 3D structural information of diverse macromolecular structures inside a given scanned sample.

To analyze the macromolecular structures in cryo-ET, two major subsequent steps need to occur. First, we need to extract the subtomograms^2^ and average those that belong to the same macromolecular class, in order to generate a high Signal-to-Noise Ratio (SNR) subtomogram for clear visualization (Zhang, 2019). Second, it is desirable to obtain the macromolecule segmentation in subtomograms to analyze the macromolecular structure parameters such as size distribution and shape. However, the macromolecular structures are highly heterogeneous and contain large quantities of subtomograms. In the past, biologists would spend large amounts of time on a set of tomograms to manually classify and segment subtomograms, but manual annotation is time-consuming and susceptible to the biases of individual biologists. Therefore, it is desirable to automatically classify the extracted subtomograms into subset of macromolecule with similar structure, and automatically generate the macromolecular segmentation.

To automate the process as well as to achieve objective analysis, deep learning methods for classification (Che et al., 2017; Xu et al., 2017; Guo et al., 2018; Zhao et al., 2018; Li et al., 2019, 2020) and segmentation (Chen et al., 2017; Liu et al., 2018; Zhou et al., 2018) have been developed for cryo-ET. Xu et al. (2017) proposed to use Inception3D network and DSRF3D network for cryo-ET subtomogram classification. Then, Chen et al. (2017) further improved the DSRF3D network with residual connection design. Guo et al. (2018) developed a cryo-ET classification model compression technique to reduce the model size while maintaining the classification performance. Zhao et al. (2018) developed a classification model visualization technique for explaining the model's attention on the classified subtomograms. For cryo-ET segmentation, Che et al. (2017) utilized independent 2D CNNs for cryo-ET tomogram components segmentation. Liu et al. (2018) built a SSN3D net for subtomogram segmentation via supervised training with large amounts of segmentation data. While previous deep learning models on cryo-ET improved the accuracy and efficiency on classification and segmentation, there are still two major bottlenecks: (1) as supervised classification methods, previous algorithms still require large amount of manually annotated training data for deep model's training, and (2) previous algorithms need to be trained again to apply to a new dataset of different classes. The open question is: Is it possible to design a generalizable cryo-ET subtomogram classification model that requires only a small reference dataset (such as one manually picked sample in each class) and match the given subtomogram to a reference class, while performing generalizable subtomogram segmentation?

Inspired by one-shot learning models which aim to learn information about object categories from one, or only a few training images (Fe-Fei et al., 2003; Koch et al., 2015), In this work, we develop a Cryo-ET One-Shot Network (COS-Net) that is able to (1) classify macromolecular structure using only a very small amount of samples, (2) simultaneously segment structural regions in a subtomogram based on the classification network, and (3) be readily and directly applied to classify and segment novel structures without needing to be re-trained. Using our COS-Net, biologists can classify and segment thousands of subtomograms by only manually picking a few representative subtomograms as support classes. When there is a need to classify new subtomogram datasets with novel structures, the support classes can be readily changed to accommodate without the need to train the model again. Moreover, unlike previous one-shot learning and few-shot learning algorithms that only address the classification task, our COS-Net can generate both classification and 3D segmentation with application in 3D imaging data of cryo-ET.

Our COS-Net is a Siamese network with pairs of volume encoders, volume decoders, and feature encoders. Given a support set of subtomograms and a target subtomogram, volume encoders first extract the volume's feature presentations. Then, the feature encoders transform the feature presentations for the next stage: one-shot learning. In the meantime, the volume decoders decode the feature presentations to generate the coarse attention/segmentation of the subtomograms. Our COS-Net with additional attention guidance from segmentation information allows better feature embedding for one-shot learning, and thus could provide better one-shot classification performance. During the test stage, we also developed a customized subtomogram processing pipeline to refine the coarse attention/segmentation from COS-Net based on 3D Conditional Random Field (3D-CRF) (Krähenbühl and Koltun, 2011). Our experimental results demonstrated that our method can effectively classify observed or novel macromolecular structures and produce accurate segmentation mask.

## 2. Methods

**Figure 1:**
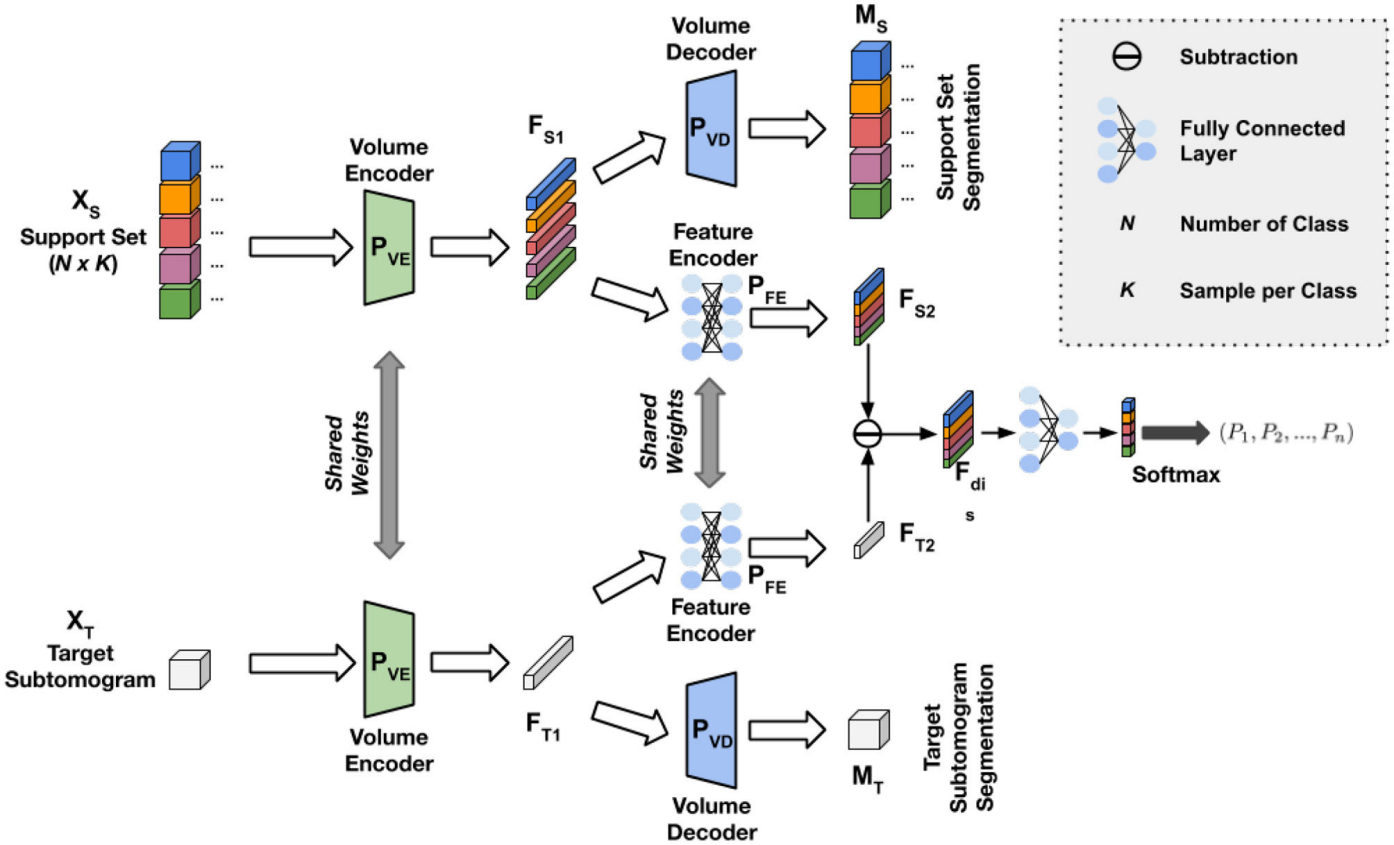
Illustration of our **C**ryo-ET **O**ne-**S**hot **Net**work (**COS-Net**) structure. The data input consists of subtomogram support set and target subtomogram. The network consists of pairs of volume encoders $P_{VE}$, volume decoder $P_{VD}$, and feature encoder $P_{FE}$ with details illustrated in Figure 2.

**Figure 2:**
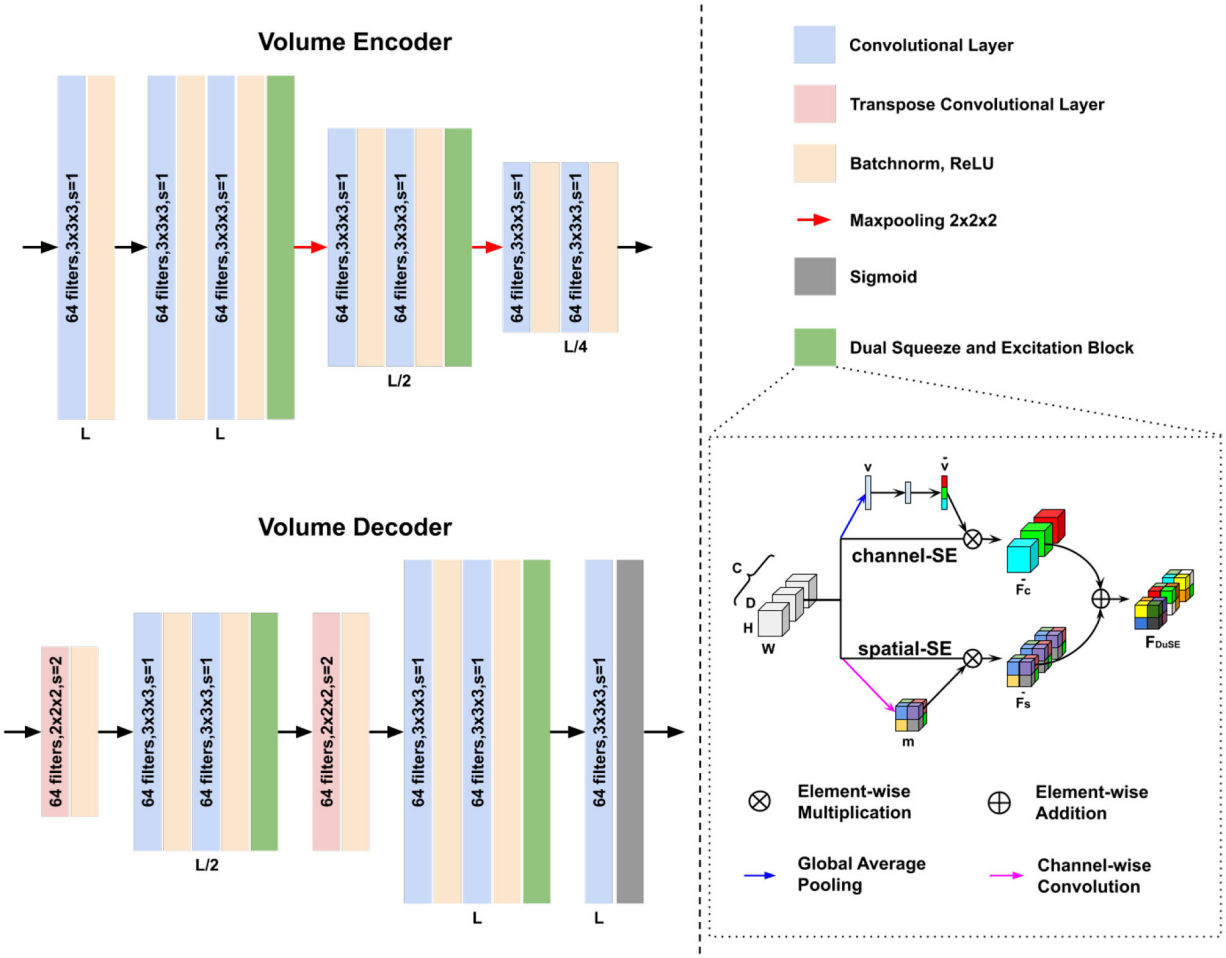
Architectures of our volume encoder and volume decoder in Figure 1. The **Du**al **S**queeze-and-**E**xcitation (**DuSE**) block is illustrated on the bottom right.

The general structure of our COS-Net is shown in Figure 1. The COS-Net is a Siamese network with two encoding-decoding streams. First, each stream consists of one volume encoder, one volume decoder, and one feature encoder. The volume encoders, volume decoders, and feature encoders shared weights between the dual streams. The design of our volume encoders, volume decoders, and feature encoders are illustrated in Figure 2 and are discussed in detail in our next section. Denoting the input for the upper stream as *X*_*S*_ that is our support set with dimensions of *N* × *K*, where *N* is the number of classes and *K* is sample per class, support set *X*_*S*_ consists of *N* classes of macromolecules with *K* samples per class. In our one-shot learning scheme, *K* = 1. The upper volume encoder takes the support set *X*_*S*_ as input and generates the latent representation of the support set with:

(1) $$\begin{array}{r} F_{S_{1}}=P_{VE}\left(X_{S}\right) \end{array}$$

where *F*_*S*_1__ is the latent representation of the support set *X*_*S*_ and $P_{VE}$ is the volume encoder function. Then, the support set's latent representations *F*_*S*_1__ are simultaneously fed into the volume decoder $P_{VD}$ and feature encoder $P_{FE}$:

(2) $$\begin{array}{r} M_{S}=P_{VD}\left(F_{S_{1}}\right) \end{array}$$

(3) $$\begin{array}{r} F_{S_{2}}=P_{FE}\left(F_{S_{1}}\right) \end{array}$$

where *M*_*S*_ is the predicted segmentation of the support set, and *F*_*S*_2__ is the feature for next stage one-shot learning. Similarly, denoting the input for the lower stream as *X*_*T*_ that is our target set with dimensions of 1 × *K*, target set *X*_*T*_ consists of 1 classes of macromolecules with *K* samples per class. In our one-shot learning scheme, *K* = 1. Similarly, the same volume encoder $P_{VE}$ takes the target set *X*_*T*_ as input and generates the latent representation of the target set with:

(4) $$\begin{array}{r} F_{T_{1}}=P_{VE}\left(X_{T}\right) \end{array}$$

where *F*_*T*_1__ is the latent representation of the target set *X*_*T*_. Then, the target set's latent representations *F*_*T*_1__ are simultaneously fed into the shared weights volume decoder $P_{VD}$ and feature encoder $P_{FE}$:

(5) $$\begin{array}{r} M_{T}=P_{VD}\left(F_{T_{1}}\right) \end{array}$$

(6) $$\begin{array}{r} F_{T_{2}}=P_{FE}\left(F_{T_{1}}\right) \end{array}$$

where *M*_*T*_ is the predicted segmentation of the target set, and *F*_*T*_2__ is the feature for next stage one-shot learning. Given the features *F*_*S*_2__ from support set and the features *F*_*T*_2__ from target set, we compute the L1 distance between the features to calculate the similarity between the support set features *F*_*S*_2__ and the target set features *F*_*T*_2__ with:

(7) $$\begin{array}{r} F_{dis}=|F_{S_{2}}-F_{T_{2}}| \end{array}$$

where *F*_*dis*_ is the feature distance. *F*_*dis*_ is then input into a fully connected layer followed by a softmax function:

(8) $$\begin{array}{r} F_{out}=softmax\left(P_{final}\left(F_{dis}\right)\right) \end{array}$$

where *F*_*out*_ is the final output with one-shot prediction indicating that the target data matches with which specific class in the support set.

**Sub-networks Design:** We use a 512 × 512 fully connected layer as our feature encoder. The volume encoder and decoder design are shown in Figure 2. Our volume encoder and volume decoder consist of three level of 3D convolution layers. Unlike conventional convolutional encoder and decoder, we concatenate a Dual Squeeze-and-Excitation (DuSE) block at each level's output in order to re-calibrate the features channel-wise and spatial-wise. More specifically, as illustrated in Figure 2 bottom right, our DuSE block contains two 3D Squeeze-and-Excitation branches for spatial-Squeeze-channel-Excitation (scSE) and channel-Squeeze-spatial-Excitation (csSE), respectively (Hu et al., 2018; Roy et al., 2018).

For scSE, we spatial-wise squeeze the input feature map using global average pooling, where the feature map is formulated as *F* = [*f*_1_, *f*_2_, …, *f*_*C*_] here with $f_{n}\in {\mathbb{R}}^{H\times W\times D}$ denoting the individual feature channel. We flatten the global average pooling output, generating *v* ∈ ℝ^*C*^ with its *z*-th element:

(9) $$\begin{array}{r} v_{z}=\frac{1}{H\times W\times D}\overset{H}{\underset{i}{\sum }}\overset{W}{\underset{j}{\sum }}\overset{D}{\underset{k}{\sum }}f_{z}\left(i,j,k\right) \end{array}$$

where vector *v* embeds the spatial-wise global information. Then, *v* is feed into two fully connected layers with weights of $w_{1}\in {\mathbb{R}}^{\frac{C}{2}\times C}$ and $w_{2}\in {\mathbb{R}}^{C\times \frac{C}{2}}$, producing the channel-wise calibration vector:

(10) $$\begin{array}{r} \hat{v}=\sigma \left(w_{2}\eta \left(w_{1}v\right)\right) \end{array}$$

where η and σ are the ReLU and Sigmoid activation function, respectively. The calibration vector is applied to the input feature map using channel-wise multiplication, namely channel-Excitation:

(11) $$\begin{array}{r} {\hat{F}}_{sc}=\left[f_{1}{\hat{v}}_{1},f_{2}{\hat{v}}_{2},\ldots ,f_{C}{\hat{v}}_{C}\right] \end{array}$$

where ${\hat{v}}_{i}$ indicates the importance of the *i*-th feature channel and lies in [0, 1]. With scSE embedded into our network, the calibration vector adaptively learns to emphasize the important feature channels while playing down the others.

In csSE, we formulate our feature map as *F* = [*f*^1, 1, 1^, …, *f*^*i, j, k*^, …, *f*^*H, W, D*^], where *f*^*i, j, k*^ ∈ ℝ^*C*^ indicates the feature at spatial location (*i, j, k*) with *i* ∈ {1, …, *H*}, *j* ∈ {1, …, *W*}, and *k* ∈ {1, …, *D*}. We channel-wise squeeze the input feature map using a convolutional kernel with weights of $w_{3}\in {\mathbb{R}}^{1\times 1\times 1\times C\times 1}$, generating a volume tensor *m* = *w*_3_ ⊛ *F* with *m* ∈ ℝ^*H*×*W*×*D*^. Each *f*^*i, j, k*^ is a linear combination of all feature channel at spatial location (*i, j, k*). Then, the spatial-wise calibration volume that lies in [0, 1] and can be written as:

(12) $$\begin{array}{r} \hat{m}=\sigma \left(m\right)=\sigma \left(w_{3}⊛F\right) \end{array}$$

where σ is the Sigmoid activation function. Applying the calibration volume to the input feature map, we have:

(13) $$\begin{array}{r} {\hat{F}}_{cs}=\left[f^{1,1,1}{\hat{m}}^{1,1,1},\ldots ,f^{i,j,k}{\hat{m}}^{i,j,k},\ldots ,f^{H,W,D}{\hat{m}}^{H,W,D}\right] \end{array}$$

where calibration parameter of ${\hat{m}}^{i,j,k}$ provides the relative importance of a spatial information of a given feature map. Similarly, with csSE embedded into our network, the calibration volume learns to stress the most important spatial locations while ignores the irrelevant ones.

Finally, channel-wise calibration and spatial-wise calibration are combined via element-wise addition: $F_{DuSE}={\hat{F}}_{sc}+{\hat{F}}_{cs}$. With the two SE branch fusion, feature at (*i, j, k, c*) possess high activation only when it receives high activation from both scSE and csSE. Our DuSE encourages the networks to re-calibrate the feature map such that more accurate and relevant feature map can be learned.

**Training Strategy and Losses:** We design a customized training strategy to train our COS-Net, such that the training procedure matches the inference at test time. Specifically, two support set are randomly generated during the training procedure. Within *N* classes, the same *n* classes are randomly sampled for each support set. 1 subtomogram is randomly sampled from these classes to form a *n-way-1-shot* scheme. The ground-truth one-shot classification label is generated by matching the class labels from the two support set, i.e., 1 for matched class label and 0 for unmatched class label.

Our training loss consists of two parts, including a Binary Cross Entropy (BCE) loss for one-shot classification learning and a Dice Similarity Coefficient (DSC) loss for one-shot segmentation. Denoting the ground-truth one-shot classification label as *F*_*gt*_, the BCE loss can be written as:

(14) $$\begin{array}{r} L_{bce}=-F_{gt}log\left(F_{out}\right)-\left(1-F_{gt}\right)log\left(1-F_{out}\right) \end{array}$$

Denoting the ground-truth subtomogram segmentation for the two support set as *M*_*gt*1_ and *M*_*gt*2_, the segmentation loss can be written as:

(15) $$\begin{array}{r} L_{dsc}=2-\frac{2\times |M_{{gt}_{1}}\cap M_{S_{1}}|}{\left|M_{{gt}_{1}}|+|M_{S_{1}}\right|}-\frac{2\times |M_{{gt}_{2}}\cap M_{S_{2}}|}{\left|M_{{gt}_{2}}|+|M_{S_{2}}\right|} \end{array}$$

where *M*_*S*_1__ and *M*_*S*_2__ are the predicted segmentation from COS-Net. The total loss thus can be formulated as:

(16) $$\begin{array}{r} L_{tot}=L_{dsc}+L_{bce} \end{array}$$

In testing, one of the support sets during training can be replaced with the target subtomogram for direct inference.

**Attention-guided Segmentation:** The segmentation predicted from COS-Net is a probability distribution, which is used for guiding our final segmentation. Specifically, the volume decoder's output is a probability distribution ranging between 0 and 1. We use a 3D Conditional Random Field (CRF) to refine and generate the final 3D subtomogram segmentation. The CRF aims to optimize the following objective function:

(17) $$\begin{array}{r} E\left(x\right)=\underset{i}{\sum }\psi_{u}\left(x_{i}\right)+\underset{i,j}{\sum }\psi_{p}\left(x_{i},x_{j}\right) \end{array}$$

where ψ_*u*_ is the unary potential that encourages the CRF output to be loyal to the probability distribution from the COS-Net. ψ_*p*_ is the pairwise potential between label on voxel *i* and *j* and can be expanded as:

(18) $$\begin{array}{l} \psi_{p}=\mu (x_{i},x_{j})[w_{1}exp\left(-\frac{|p_{i}-p_{j}{|}^{2}}{2\sigma_{\alpha }^{2}}-\frac{|I_{i}-I_{j}{|}^{2}}{2\sigma_{\beta }^{2}}\right)\\ \;+w_{2}exp\left(-\frac{|p_{i}-p_{j}{|}^{2}}{2\sigma_{\gamma }^{2}}\right)] \end{array}$$

where μ(*x*_*i*_, *x*_*j*_) is the compatibility transformation and depends on the labels *x*_*i*_ and *x*_*j*_ such that μ(*x*_*i*_, *x*_*j*_) = 1 if *x*_*i*_≠*x*_*j*_, and 0 otherwise. *I*_*i*_ and *I*_*j*_ are the intensity value at voxel location *i* and *j*. *p*_*i*_ and *p*_*j*_ are the spatial coordinates of voxel *i* and *j*. *w*_1_, *w*_2_, σ_α_, σ_β_, and σ_γ_ are learnable parameters for CRF. This term penalizes pixels with similar position *p* and intensity *x* but with different label.

## 3. Experiments and Results

### 3.1. Data Preparation

We prepared a realistically simulated dataset with known macromolecular structures by reconstructing the tomographic image using the projection images (Pei et al., 2016). The limiting factors of cryo-ET, such as noise, missing wedge, and electron optical factors (Modulation Transfer Function, Contrast Transfer Function) were all properly included. The simulation process mimicked the experimental cellular sample imaging condition and tomographic reconstruction process. We took into account the randomness of macromolecule structural poses. The packed volume containing macromolecular structures were projected to a series of 2D projection images with specified tilt angle steps. The resulting projection images were convolved to include optical factors and then back-projected to obtain the reconstructed 3D simulated tomogram. 22 distinct macromolecular structures are chosen from the Proterin Databank (PDB) with their PDB ID information (Berman et al., 2000) of atomic coordinates and connectivity, and secondary structure assignments. We choose very representative macromolecules such as ribosome (4V4Q), proteasome (3DY4), and RNA polymerase (2GHO), which are well-studied due to their abundance and importance in cellular functions. Each simulated tomogram of 600 × 600 × 300 voxels contains 10,000 randomly distributed macromolecules. Given the true position of these macromolecules inside tomograms, we collected 5,835 subtomograms of size 32 × 32 × 32, belonging to 22 structural classes. The dataset with 22 distinct classes was split into a training set with 14 classes and a test set with 8 classes. Three datasets with different levels of signal-to-noise ratio (SNR) were used, including SNR = ∞, SNR = 1, 000, and SNR = 0.5.

### 3.2. Classification Results

Table 1 summarizes the one-shot classification performance with different sub-network setup. We evaluated the one-shot classification accuracy under different noise level and various one-shot training schemes. First, comparing the COS-Net with and without volume decoder for guiding the one-shot classification, with volume decoder can significantly improve the classification accuracy for sub-networks with or without DuSE block. For example, using the SNR = 1, 000 dataset, the 2way-1shot COS-Net with DuSE improve the accuracy from 0.928 to 0.939 by adding the volume decoder. Second, comparing the COS-Net with and without DuSE block, adding DuSE block to volume encoder/decoder can also improve the classification accuracy. However, the classification accuracy decreases as the SNR decreases, due to the structural details being degraded by noise. Meanwhile, the classification accuracy also decreases as the number of classes (way) increase.

**Table 1:** The one-shot classification accuracy on three dataset with three different SNR levels.

**Data**	**Networks**	**2way-1shot**	**4way-1shot**	**6way-1shot**	**8way-1shot**
SNR:∞	SCNN w/o Decoder	0.931	0.763	0.613	0.595
	SCNN w Decoder	0.945	0.798	0.663	0.636
	DuSE-SCNN w/o Decoder	0.934	0.772	0.618	0.603
	DuSE-SCNN w Decoder	0.957	0.831	0.672	0.646
	SCNN w/o Decoder	0.923	0.698	0.493	0.473
	SCNN w Decoder	0.935	0.706	0.493	0.473
SNR:1000	DuSE-SCNN w/o Decoder	0.928	0.701	0.504	0.479
	DuSE-SCNN w Decoder	0.939	0.718	0.534	0.513
	SCNN w/o Decoder	0.812	0.599	0.501	0.387
	SCNN w Decoder	0.824	0.616	0.502	0.399
SNR:0.5	DuSE-SCNN w/o Decoder	0.821	0.614	0.510	0.391
	DuSE-SCNN w Decoder	0.829	0.628	0.513	0.403

*2way-1shot, 4way-1shot, 6way-1shot, and 8way-1shot learning scenarios are included. The highest accuracy for each learning scenario is marked in blue*.

### 3.3. Segmentation Results

**Figure 3:**
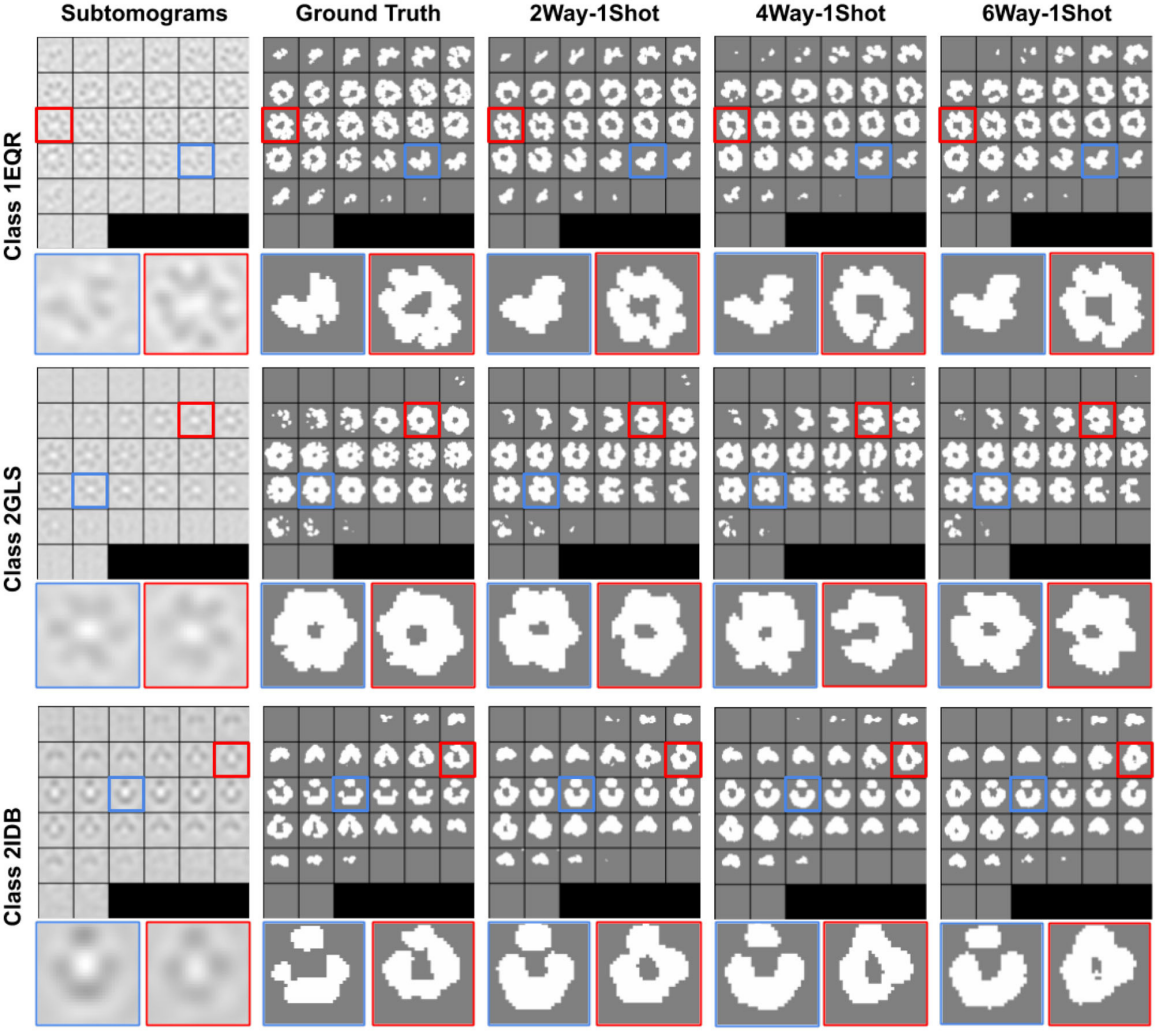
Illustration of segmentation results on all three test classes using COS-Net with DuSESCNN. The macromolecule PDB ID is indicated for each classes on the left. The ground truth segmentation (second column) is compared against COS-Net with 2way-1shot, 4way-1shot, 6way-1shot scenarios from second to fifth column. The enlarged images on selected 2D slices are visualized at the bottom.

**Figure 4:**
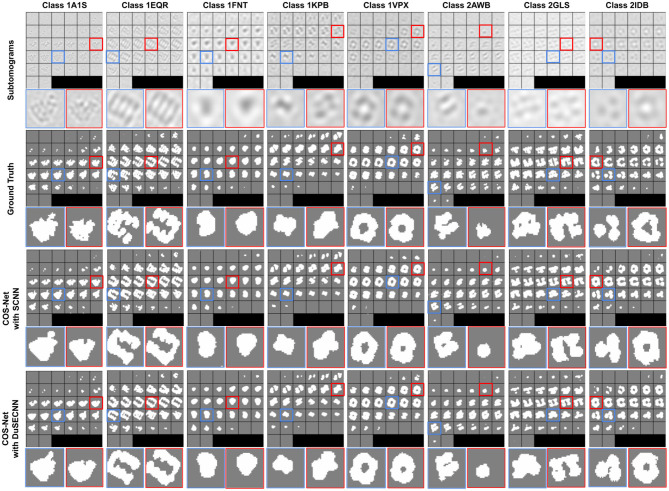
Illustration of segmentation results on all eight test classes using 2way-1shot. The macromolecular PDB ID is indicated for each classes on the top. The ground truth segmentation (second row) is compared against COS-Net with SCNN (third row) and COS-Net with DuSESCNN (fourth row). The enlarged images on selected 2D slices are visualized at the bottom.

The segmentation performance of our attention-guided segmentation is evaluated using the same test set as in the classification section based on DSC:

(19) $$\begin{array}{r} DSC=\frac{2\times |M_{gt}\cap M_{pred}|}{\left|M_{gt}|+|M_{pred}\right|} \end{array}$$

where *M*_*pred*_ is our generated segmentation, and *M*_*gt*_ is the ground-truth segmentation. Segmentation results with different training schemes on SNR = 1, 000 dataset are visualized in Figure 3. As we can see, our method can generate accurate 3D segmentation that does not rely on unseen classes' pixel-level or image-level training data. It is also worth notice that our method can achieve robust and consistent segmentation performance over different way one shot learning schemes. Besides, a comparison of segmentation results with and without DuSE block on eight different macromolecule classes is visualized in Figure 4. While segmentation with DuSE block does not significantly outperforms segmentation without DuSE block, they both produce reasonable segmentation of macromolecules.

The quantitative results using SNR = 1, 000 and SNR = ∞ datasets are summarized in Tables 2, 3, respectively. As we can observe, for all 8 unseen classes, our COS-Net is able to generate reasonable 3D segmentation. For SNR = ∞ data, the DSC of our COS-Net with DuSE are all > 0.92 for all classes, indicating accurate 3D macromolecule segmentation. For SNR = 1, 000, the DSC of COS-Net with DuSE are > 0.84. The decrease in segmentation performance is due to the increased noise level that degrades the macromolecule structure details. However, as illustrated in Figure 3, our COS-Net can still generate reasonable 3D segmentation for unseen classes.

**Table 2:** The segmentation results for all eight test classes on SNR = 1,000 dataset.

**SCNN**	**1A1S**	**1BXR**	**1EQR**	**1F1B**	**1FNT**	**1GYT**	**1KPB**	**1LB3**
2way-1shot	0.84 ± 0.07	0.85 ± 0.02	0.86 ± 0.02	0.87 ± 0.01	0.89 ± 0.01	0.84 ± 0.01	0.88 ± 0.01	0.83 ± 0.01
4way-1shot	0.84 ± 0.07	0.85 ± 0.02	0.86 ± 0.02	0.87 ± 0.01	0.90 ± 0.01	0.85 ± 0.01	0.88 ± 0.01	0.84 ± 0.02
6way-1shot	0.85 ± 0.08	0.85 ± 0.02	0.85 ± 0.02	0.87 ± 0.01	0.89 ± 0.01	0.84 ± 0.01	0.87 ± 0.01	0.84 ± 0.01
8way-1shot	0.85 ± 0.07	0.84 ± 0.02	0.86 ± 0.02	0.87 ± 0.01	0.90 ± 0.01	0.85 ± 0.01	0.88 ± 0.01	0.83 ± 0.01
**DuSE-SCNN**	**1A1S**	**1BXR**	**1EQR**	**1F1B**	**1FNT**	**1GYT**	**1KPB**	**1LB3**
2way-1shot	0.85 ± 0.08	0.85 ± 0.02	0.86 ± 0.02	0.87 ± 0.01	0.90 ± 0.01	0.85 ± 0.01	0.88 ± 0.01	0.85 ± 0.01
4way-1shot	0.85 ± 0.07	0.85 ± 0.02	0.85 ± 0.02	0.87 ± 0.01	0.90 ± 0.01	0.85 ± 0.01	0.88 ± 0.01	0.85 ± 0.01
6way-1shot	0.85 ± 0.08	0.85 ± 0.02	0.86 ± 0.02	0.87 ± 0.01	0.90 ± 0.01	0.85 ± 0.01	0.88 ± 0.01	0.85 ± 0.02
8way-1shot	0.84 ± 0.08	0.85 ± 0.01	0.86 ± 0.02	0.87 ± 0.01	0.90 ± 0.01	0.85 ± 0.01	0.88 ± 0.01	0.85 ± 0.02

*The mean±standard deviation DSC are reported in the table. 2way-1shot, 4way-1shot, 6way-1shot, and 8way-1shot learning scenarios are reported at different rows. The macromolecular PDB ID is indicated for each classes*.

**Table 3:** The segmentation results for all eight test classes on SNR = ∞ dataset.

**SCNN**	**1A1S**	**1BXR**	**1EQR**	**1F1B**	**1FNT**	**1GYT**	**1KPB**	**1LB3**
2way-1shot	0.92 ± 0.08	0.94 ± 0.03	0.98 ± 0.02	0.97 ± 0.02	0.97 ± 0.03	0.95 ± 0.03	0.96 ± 0.01	0.97 ± 0.02
4way-1shot	0.92 ± 0.08	0.95 ± 0.03	0.98 ± 0.02	0.97 ± 0.02	0.97 ± 0.02	0.95 ± 0.03	0.96 ± 0.03	0.97 ± 0.03
6way-1shot	0.92 ± 0.08	0.94 ± 0.04	0.98 ± 0.01	0.96 ± 0.02	0.97 ± 0.02	0.95 ± 0.03	0.96 ± 0.01	0.96 ± 0.02
8way-1shot	0.92 ± 0.08	0.94 ± 0.03	0.98 ± 0.02	0.96 ± 0.02	0.97 ± 0.02	0.95 ± 0.02	0.96 ± 0.02	0.96 ± 0.02
**DuSE-SCNN**	**1A1S**	**1BXR**	**1EQR**	**1F1B**	**1FNT**	**1GYT**	**1KPB**	**1LB3**
2way-1shot	0.92 ± 0.08	0.94 ± 0.03	0.98 ± 0.02	0.97 ± 0.02	0.97 ± 0.02	0.95 ± 0.03	0.96 ± 0.02	0.97 ± 0.02
4way-1shot	0.93 ± 0.07	0.96 ± 0.02	0.98 ± 0.01	0.97 ± 0.02	0.97 ± 0.02	0.95 ± 0.03	0.96 ± 0.02	0.97 ± 0.02
6way-1shot	0.92 ± 0.08	0.95 ± 0.03	0.98 ± 0.02	0.97 ± 0.02	0.97 ± 0.02	0.95 ± 0.02	0.96 ± 0.02	0.96 ± 0.02
8way-1shot	0.92 ± 0.07	0.94 ± 0.03	0.98 ± 0.02	0.96 ± 0.02	0.97 ± 0.02	0.95 ± 0.02	0.96 ± 0.02	0.96 ± 0.03

*The mean±standard deviation DSC are reported in the table. 2way-1shot, 4way-1shot, 6way-1shot, and 8way-1shot learning scenarios are reported at different rows. The macromolecular PDB ID is indicated for each classes*.

## 4. Discussion and Conclusion

In this work, we developed a one-shot learning framework for cryo-ET where simultaneous classification and segmentation can be performed for seen or unseen macromolecule subtomograms. Specifically, we developed a COS-Net to learn the class matching between a support set consisting of multiple classes with only 1 sample per class and a target subtomogram. In COS-Net, the segmentation attention is utilized to better guide the one-shot classification. In the mean time, the volume decoder of COS-Net allows us to generate the coarse segmentation of the macromolecule in the subtomogram. Then, 3D CRF is utilized to refine the 3D macromolecule segmentation from COS-Net.

We demonstrated the successful application of our COS-Net on a cryo-ET dataset consisting of 22 macromolecule classes. First, our method demonstrated accurate one-shot classification performance over dataset with different noise levels. Even with SNR as low as 0.5, the classification accuracy is over 0.8 in a 2way-1shot classification scheme. As compared to previous supervised cryo-ET classification methods with classification accuracy of about 0.9, our method is able to achieve comparable performance without using large-scale high-quality labeled data (Liu et al., 2018; Che et al., 2019). Second, our method can produce high-quality 3D segmentation for unseen macromolecules under different one-shot classification schemes. As we can observe in Table 3, our COS-Net can produce 3D segmentation with DSC> 0.84 on all test macromolecules over all one-shot schemes. As compared to previous supervised segmentation methods, our segmentation performance is comparable to these supervised cryo-ET segmentation models with DSC of about 0.88, which require segmentation ground truth on seen macromolecule classes for training (Liu et al., 2018; Che et al., 2019). Therefore, our method provides a solution of both accurate classification and segmentation for unseen macromolecule classes.

The presented work can potentially be further improved from the following perspectives. First of all, the classification accuracy decreases as the number of classes in the support set increases. As more classes are involved in the class matching procedure and only one sample is used for each classes, the classification difficulty will naturally increase. However, our COS-Net can be extended from one-shot to few-shot if more samples are available for each class, and this strategy could potentially improve the classification accuracy. Moreover, the macromolecule alignment is not considered in the current one-shot classification pipeline. The macromolecule in the support set and target set may not be aligned, i.e., they have different orientations before feeding into our network, which could potentially decrease the classification accuracy. Subtomogram pre-processing by alignment of macromolecule in subtomograms could potentially further improve our classification accuracy and will be a focus in our future work (Lü et al., 2019; Zeng and Xu, 2020). Second, the cryo-ET imaging data is reconstructed from limited angle conditions. The subtomogram image quality could be degraded by the limited angle reconstruction artifacts and potentially impact the downstream COS-Net's performance. Deep learning based limited angle reconstruction algorithms could be incorporated to mitigate these artifacts and potentially further improve our performance (Zhou et al., 2019, 2020). Third, our study is performed based on realistically simulated cryo-ET dataset with sufficient amounts of macromolecule classes for one-shot learning studies. Currently, real cryo-ET data does not provide sufficient amounts of classes for one-shot learning studies, and we will include it in our future studies.

In summary, we developed a COS-Net for one-shot classification and segmentation in cryo-ET, which enables the classification and segmentation for unseen macromolecules in the wild. We believe our algorithm is an important step toward the large-scale and systematic *in situ* analysis of macromolecular structure in single cells captured by cryo-ET.

## Data Availability Statement

The raw data supporting the conclusions of this article will be made available by the authors, without undue reservation.

## Author Contributions

BZ: conceptualization, methodology, software, visualization, validation, formal analysis, and writing original draft. HY: methodology, software, visualization, validation, formal analysis, and writing original draft. XZ: conceptualization, methodology, and writing—review and editing. XY: software, visualization, validation, and formal analysis. JZ: writing—review and editing, and supervision. MX: conceptualization, methodology, writing—review and editing, and supervision. All authors contributed to the article and approved the submitted version.

## Conflict of Interest

The authors declare that the research was conducted in the absence of any commercial or financial relationships that could be construed as a potential conflict of interest.

## Footnotes

^1^At their original locations.

^2^Small cubic subvolumes containing one macromolecular structure.
